# Treatment outcomes of tuberculosis patients in a Directly Observed Treatment Short course (DOTS) Referral Centre in Delta State, Nigeria: a five-year review (2012 – 2016)

**DOI:** 10.4314/ahs.v22i2.20

**Published:** 2022-06

**Authors:** Nyemike Simeon Awunor, Innocent Osi Alenoghena, Alexander Akpodiete

**Affiliations:** 1 Department of Community Medicine, Delta State University, Abraka, Nigeria; 2 Department of Community Health, Ambrose Alli University, Ekpoma, Edo State Nigeria; 3 Delta State Tuberculosis and Leprosy Referral Centre, Eku, Nigeria

**Keywords:** Tuberculosis, DOTS, Treatment Outcome, TB/HIV Co-infection, Delta State

## Abstract

**Introduction:**

The objective of this study is to observe the trend in treatment outcomes and identify determinants of treatment success among patients recruited into care through the DOTS strategy.

**Methodology:**

A retrospective record review of tuberculosis patients (2012–2016) was carried out at the Tuberculosis and Leprosy Referral Centre, Eku, Delta State, Nigeria.

**Results:**

Records of four hundred and twenty five (425) tuberculosis patients under DOTS were reviewed over five years. The highest number of cases under treatment, 102 (24.0%), was recorded in 2013. The mean age (SD) of patients was 37.3 (±16.5) years, majority of the patients were male (62.4%) and 18% had TB/HIV co-infection. Treatment outcomes of patients were cured (53.4%), completed (27.8%), died (6.8%), failed (2.4%), lost to follow up (4.9%), transferred out (1.2%) and not evaluated (3.5%). Over all, treatment success rate was 81.2% with a trend of 88.7% (2012), 87.3% (2013), 85.9% (2014), 65.0% (2015) and 65.8% (2016) respectively. Patient characteristics were not associated with treatment success.

**Conclusion:**

The treatment success rate was high and in line with the national recommendation of 80% and above. The trend showed a reduction in number of new cases enrolled into the DOTS programme, reduction in success rate with a concomitant increase in loss to follow up. There was no association between patient characteristics and TB treatment success. System strengthening on patient follow up, community health education and treatment adherence is recommended.

## Introduction

Tuberculosis (TB) is an infectious disease that substantially contributes to the global burden of communicable disease morbidity and mortality. It forms part of the big threes of HIV/AIDS, tuberculosis and malaria that are specifically spelt out for global epidemiological control within the framework of the United Nation's sustainable development goals (SDG). 1 In 2015, there were an estimated 10.4 million new (incident) TB cases worldwide, amongst which were 5.9 million (56%) men, 3.5 million (34%) women and 1.0 million (10%) children. People living with HIV accounted for 1.2 million (11%) of all new TB cases. Six countries accounted for 60% of the new cases namely India, Indonesia, China, Nigeria, Pakistan and South Africa. 1

Global progress depends on major advances in TB prevention and care in these countries. Worldwide, the rate of decline in TB incidence remain at only 1.5% from 2014 to 2015.^2^ This needs to accelerate to a 4–5% annual decline by 2020 to reach the first milestones of the End TB Strategy.^2^

Nigeria is categorised as a high TB burden country, added to its challenges as regard effective HIV/AIDS prevention and control, which runs a national TB prevention and control programme. 1,2 National TB control can been affected by such problematic issues as weak surveillance systems, HIV co-infection, problems of non-adherence to medication, treatment default, loss to follow up and an increasing incidence of TB-drug resistance.^1^,^3^ The directly observed treatment short course (DOTS) strategy has over the years sought to tackle the above listed challenges. Peculiarities in the public health operating systems of various countries; economic, social and demographic characteristics can also play a major role in determining the level of success of this evidence based strategy in various climes.^4^,^5^ Outcomes of interest under the programme management guidelines include cure, treatment completed, treatment failure, death, default and transferred out.^6^ Some studies in Nigeria have reported TB treatment success rates ranging from 58.6% to 86.4%.^5^,^7^–^9^

This underscores the fact that determinants of treatment success could vary from state to state, country to country or region to region. 7,9–13 Studies to identify the determinants of treatment outcomes are relevant especially in low and middle income countries as this would improve the monitoring and evaluation of on-going national TB control programmes. 4,5,14,15

According to the National Tuberculosis, Leprosy and Buruli Ulcer Management and Control Guidelines, the Targets for TB control in Nigeria are to detect at least 70% of the estimated all forms of TB cases by 2020, to achieve a treatment success rate of at least 90% for all new bacteriologically confirmed TB cases by 2020 and to eliminate TB as a public health problem (<= 1/1,000,000 population) by 2050. 6

The objective of this study is to observe the trend in treatment outcomes and identify determinants of treatment success among patients recruited into care through the DOTS strategy over a five-year period (2012–2016) at the Tuberculosis and Leprosy Referral Centre, Eku, Delta State, Nigeria.

## Methods

### Study Area

Tuberculosis and Leprosy Referral Hospital, Eku in Ethiope East Local Government Area, Delta State, Southern Nigeria. The treatment regimen of the centre has two categories (CAT), one of 6 months (CAT I) mainly for new cases and another (CAT II) for cases less likely to respond to normal regimen like previously failed cases.

### Study Population and Study Design

All cases of tuberculosis treated in the facility from January 2012 to December 2016 and using a retrospective record review.

### Sample size

Using the Cochran formula (n=z2 pq/d2) with a success rate, p= 0.85 5 and q=1-p, allowing for an error level of 0.05; and allowing for 10% adjustment for incomplete responses the minimum sample size determined for this study was 216. However, four hundred and twenty-five patient case records were used for this study.

### Data Collection and Study Tools

The tuberculosis and leprosy referral centre's patient's case notes provided the information from which a clinical report form specially designed to extract information from the case notes was used for data collection.

### Standard Tb Case And Treatment Outcome Definitions

The following TB cases and treatment outcomes were defined according to the National Tuberculosis, Leprosy and Buruli Ulcer Management and Control Guidelines.

**New smear positive pulmonary TB:** A TB patient who had never taken treatment for TB, or who had taken anti-TB drugs for less than four weeks and had at least one sputum smear positive sample.

**Other Cases:** TB cases who were not new, relapse, treatment after failure, and treatment after loss to follow-up.

### Cured

TB patient diagnosed smear or culture positive at the beginning of treatment, who completed treatment, and whose sputum smear culture was negative in the last month of treatment and on at least one previous occasion.

### Treatment Completed

TB patient who completed treatment but without evidence of cure or failure (no record to show that sputum smear or culture results in the last month of treatment and on at least one previous occasion are negative.

### Treatment Failure

Patient diagnosed smear or culture positive at the beginning of treatment whose sputum smear or culture is positive at month 5 or later during treatment.

**Died:** TB patients who died for any reason during the course of treatment.

### Defaulter/loss to follow-up

A TB patient who had been on treatment for at least four weeks and interrupted treatment for two consecutive months or more.

Successful Treatment (good outcome): A treatment that ended up as cure or treatment completed. Unsuccessful treatment (bad outcome): A treatment that ended up in treatment default/loss to follow-up, treatment failure, or death. 6

### Data Analysis

This was done using the statistical package for scientific solutions (IBM SPSS) version 21 software. Results were presented in statements, tables and charts. Statistical test of association was done with significance set at p<0.05.

### Ethical Consideration

This research proposal was submitted to the Ethical Committee of Delta State University Teaching Hospital, Oghara and approval was secured to proceed with the study in December 2017. Institutional permission was obtained from the Medical Director of the facility.

## Results

### Demographic characteristics of patients

Records of four hundred and twenty-five (425) tuberculosis patients under DOTS were reviewed over five years. Table 1 reveals the mean age of cases, which was 37.3±16.5 years. Most of the respondents, 114 (26.8%) were within the ages of 31–40 years. A greater proportion of the respondents 265 (62.4%) were males Also, majority of them, 340 (80%) were new cases. Three hundred and eight (72.5%) of the respondents were HIV negative, 18.1% (77) were HIV positive while 9.4% (40) had unknown status. Ninety nine percent of the cases had pulmonary tuberculosis while 4 (0.9%) had extra-pulmonary tuberculosis, 2 of which sites were not stated while the other 2 were throat and supraclavicular lymph node tuberculosis.

**Table 1:** Demographic Characteristics of Respondents (n=425)

Characteristic	Frequency	Percent
**Age Group (years)**		
≤ 20	50	11.8
21–30	107	25.2
31–40	114	26.8
41–50	60	14.1
51–60	46	10.8
>60	48	11.3
**Sex**		
Male	265	62.4
Female	160	37.6
**HIV Status**		
Unknown	40	9.4
Positive	77	18.1
Negative	308	72.5
**Case Type at Recruitment**		
New case	340	80.0
Others*	85	20.0
**Site of Infection**		
Pulmonary	421	99.1
Extra Pulmonary	4	0.9

* Others- Failure=6, Defaulted=43, Relapsed=23, Transferred in=6, Unclassified=7 Mean (SD) age = 37.3 (±16.5) years

### Trend in cases and treatment outcomes of patients

**Figure 1:**
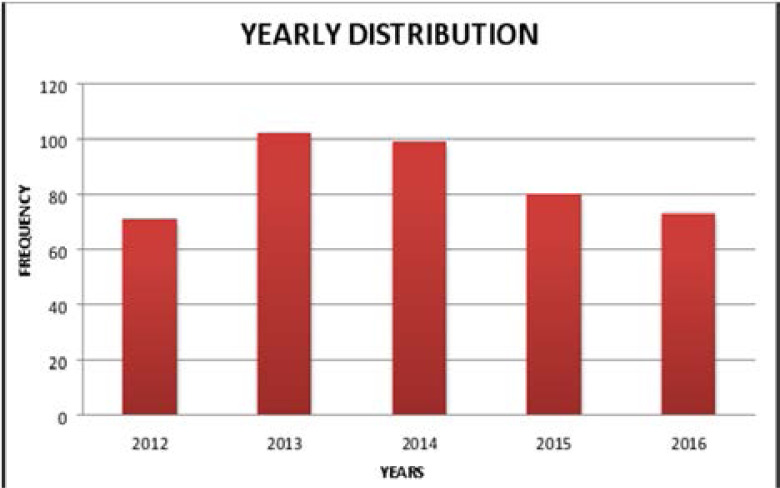
Trend in Cases Managed at the Tuberculosis Treatment Centre (2012–2016)

Figure 1 shows that a total of 425 cases of tuberculosis had been treated in the Tuberculosis and Leprosy Centre Eku for the 5-year period, with 71 (16.7%), 102 (24.0%), 99 (23.3%), 80 (18.8%), and 73 (17.2%) cases for 2012, 2013, 2014, 2015 and 2016 respectively. The trend in recruitment of cases (2012–2016) showed a peak in 2013 with a subsequent drop in the last two years of the study period.

Table 2 shows results of bacteriological test done on Sputum samples either by AFB Microscopy or Gene Xpert. “Negative” indicates that the TB Bacilli were not found and “Positive” means the bacilli were found. A patient with negative sputum result is enrolled into care if a Medical officer sees the same patient and considered the clinical presentation of the patient, assisted with radiological examination to be enough evidence to diagnose TB. “Not done” indicates if sputum was not tested or the documentation of the Sputum test was not found in the records reviewed. Of the 425 patients' file reviewed almost 127 (30%) were sputum negative or clinically diagnosed and almost 283 (67%) were bacteriologically diagnosed. Less than 15 (4%) results were not documented. Bacteriological diagnosis was more in 2015 and 2016 due to the introduction of Gene Xpert test as the first means sputum testfor TB in the Programme in Nigeria.

**Table 2:** A Table Showing the Trend of Smear Results Categorized by Year

	Smear result n (%)			Total
	
	Negative	Not Done	Positive	
2012	18 (25.4)	4 (5.6)	49 (69.0)	71 (100.0)
2013	39 (38.2)	5 (4.9)	58 (56.9)	102 (100.0)
2014	44 (44.4)	4 (4.0)	51 (51.5)	99 (100.0)
2015	13 (16.3)	2 (2.5)	65 (81.3)	80 (100.0)
2016	13 (17.8)	0 (0.0)	60 (82.2)	73 (100.0)

**Total**	**127 (29.9)**	**15 (3.5)**	**283 (66.6)**	**425 (100.0)**

Table 3 shows the outcome of patients on treatment, the data showed cured (53.4%), completed (27.8%), died (6.8%), failed (2.4%), lost to follow up (4.9%), transferred out (1.2%) and not evaluated (3.5%).

**Table 3:** Treatment Outcome of the Cases Seen in the Centre

Treatment Outcome	Frequency	Percent
Cured	227	53.4
Completed	118	27.8
Died	29	6.8
Failed	10	2.4
Lost to follow up	21	4.9
Transferred out	5	1.2
Not evaluated	15	3.5

**Total**	**425**	**100.0**

**Figure 2:**
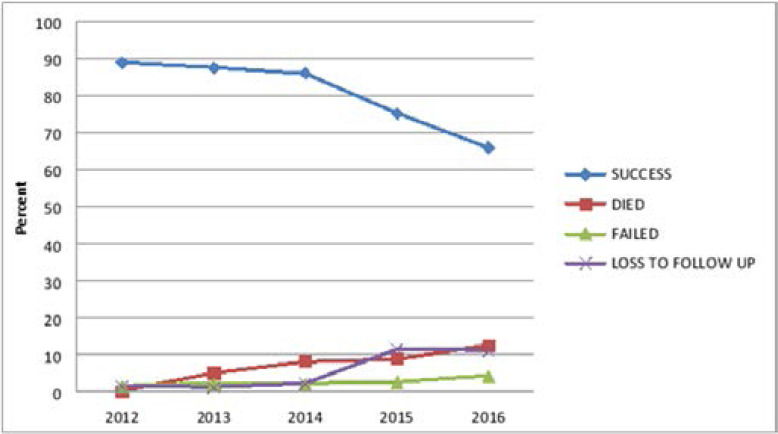
Trend in Tuberculosis Treatment Outcomes (2012–2016)

Figure 2 shows the trend in tuberculosis treatment outcomes (2012–2016). Treatment success rate (cured + completed) was 81.2% and there was a reduction in the treatment success rates in 2015 and 2016 respectively. The trend in treatment success rate was 2012 (88.7%), 2013 (87.3%), 2014 (85.9%), 2015 (65.0%) and 2016 (65.8%) respectively. The trend in treatment failure rate was 2012 (1.4%), 2013 (1.0%), 2014 (2.0%), 2015 (2.5%) and 2016 (4.1%) respectively. The trend in loss to follow up/default rate was 2012 (1.4%), 2013 (1.0%), 2014 (2.0%), 2015 (11.3%) and 2016 (11.0%) respectively.

### Determinants of treatment success in patients

Characteristics of patients and TB treatment success was assessed in table 4, none of the characteristics of age OR (95%CI); p = 1.14 (0.67–1.93); 0.604, sex OR (95%CI); p = 0.66 (0.37–1.14); 0.117 and HIV status OR (95%CI); p = 0.92 (0.47–1.87); 0.793 was associated with treatment success in TB patients.

**Table 4:** Patient Characteristics and Tuberculosis Treatment Success (n=425)

Characteristic	Treatment success, n (%)		OR (95%CI)	p-value

	Yes	No		
**Age Group (years)**				
≤ 40	222 (81.9)	49 (18.1)	1.14 (0.67–1.93)	0.604
40 and above	123 (79.9)	31 (20.1)	1	
**Sex**				
Male	209 (78.9)	56 (21.1)	0.66 (0.37–1.14)	0.117
Female	136 (85.0)	24 (15.0)	1	
**HIV Status (n=385)**				
Positive	62 (80.5)	15 (19.5)	0.92 (0.47–1.87)	0.793
Negative	252 (81.8)	56 (18.2)	1	

## Discussion

Four hundred and twenty-five 425 patients diagnosed with TB were managed in the Tuberculosis and Leprosy Referral Centre, Eku from 2012 to 2016. The characteristic profile over this period revealed a gradual reduction in new cases. Close to a fifth of all patients had TB/HIV co-infection. There was an improvement in diagnostic completeness of TB over this period. The mean age of the patients admitted was 37.3 (±16.5) years. This is similar to findings from Ikenna et al. in Niger Delta University Teaching Hospital, Okolobiri which had a mean age of 37.0 (±16.3) years. 7 Other studies have shown the highest proportion of their TB patients within that range 9,13,15 while Olarewaju et al. reports a higher mean age of 42.4 (±1.9) years among respondents.5 The sex distribution of the study population shows a higher prevalence of the disease in males with a ratio of 1.7 to 1 which is in keeping with the global ratio of 1.2 to 1 and similar to findings from previous studies by Nwachukwu et al in 3 DOTS Centres in Anambra, 16 Olarewaju et al in Ogbomoso (2008 to 2012), 5 and Onorikpori in Eku hospital (2009 to 2013).^8^

In addition, the profile of the patients reveals that four fifths of the cases were newly diagnosed cases, while about a fifth of the cases were retreatment which could have implications for treatment outcomes. Ninety nine percent of the cases had pulmonary tuberculosis while just 4 (0.9%) had extra-pulmonary tuberculosis, 2 of which sites were not stated while the other 2 were throat and supraclavicular lymph node tuberculosis. This is indeed, comparable to reports by Olarewaju et al 5 in Ogbomoso (2008 to 2012) with pulmonary of 88.5% which is in line with the fact that pulmonary tuberculosis is the predominant clinical manifestation of the disease globally.

In terms of treatment outcome over the five-year period, and considering success as a combination of treatment completed and cured, three quarter of the cases had a successful outcome. Overall the available data shows that the referral centre had met the National treatment success rate of 80% and was in line to meet the 2020 target of a success rate 90% or above. 2,6 This is consistent with the findings of Olarewaju et al 5 in Ogbomoso (2008 to 2012) who reported a success rate of 85.5% and 9.52% death and Nwachukwu et al 16 in 3 DOTS centres in Anambra with a success rate of 86.4%, Zenebe et al. in Eastern Ethiopia 10, Asfaw et al. in Ethiopia 11 and Emeirya et al. in Egypt 15 but at variance with the result of Onorikpori et al 8 in Eku hospital (2009 to 2013) with a success rate of 58.6%, Ebuenyi et al. 7, Fiseha et al. 17 and Abdulkader et al. 9

With regards to the proportion of patients that died, finding from this study at 6.8% was higher than what was seen in recent studies in Nigeria and Ethiopia 7,9,10,12,18, but less than what was observed in a few other studies 4,5. Concerning treatment failure, Zenebe et al. 10, Olarewaju et al 5 and Abdulkader et al 9 and Yakob et al. 18 reported a much lower rate when compared to the present study, however the study by Ebuenyi et al 7 in Nigeria reported a failure rate of 4.8% which was twice that of the present study. About loss to follow up, the spectrum varied when compared to the present study at 4.9% as it ranged from as low as 0.9% in the study by Olarewaju et al. 5 to as high as 18.9% by Njelita et al.^19^. A number of studies reporting within this range were also observed^4^,^7^,^9^,^10^,^12^,^18^, which suggests a wide variation in health facility practices with regards to the follow up of their DOTS patients and its implications on treatment outcomes. With regards to patients transferred out of the TB treatment centre, most studies reported rates higher than what was observed in the present study at 1.2% 5,7,9,10,16–18.

The trend in TB outcomes shows that in the last two years of the period under study, there was a drop observed in the treatment success rates while there was a steady rise in the default rate (comprising treatment failure and loss to follow up) among the TB patients. Further research into the determinants of default in these patients will be required and a qualitative enquiry would help in eliciting sociological factors influencing default among patients in low and middle income countries.

With regards to the HIV status of the patients, about three quarter of them were HIV negative, and about a fifth of them were HIV positive while a tenth of the patients had unknown status. This is not consistent with results from a previous study carried out in Abuja; with up to 47% being HIV positive and that by Ikenna et al 7 with a prevalence rate of 55.4%. Furthermore, treatment success for HIV negative and positive cases were similar in the present study as the HIV status of these patients did not significantly affect their treatment success. This is not in keeping with the results from a study by Onyebuchi et al at 20 at National Hospital Abuja in 2015 which had a very strong evidence of an association between HIV status and cure rate. Njelita et al. 19 also reported an association of treatment outcome with the age and HIV status of patients.

Factors such as age and sex of respondents were not associated with treatment success in this study. This finding is at variance with the study by Omotowo et al. 21 in Nigeria that reported sex and duration of treatment as associated with treatment success. Fiseha et al. in Ethiopia also reported an association of treatment success with age and year of enrolment of patients 17.

This study relied on secondary data and was limited in obtaining some of the socio-demographic characteristics of the cases as provision was not made for occupation, educational status, and marital status in most of the case notes in the earlier years. The records that were complete, as seen in the later years, were too few to make generalizations about the study population. There is a need to sustain the improvement in reporting seen in the later years of the period under study.

The effect of mycobacterial resistance on treatment success could not be assessed as resistance testing by the gene Xpert procedure started relatively recently in the referral facility, with reference to this study period, and reflected essentially in the 2016 cases and in just a few cases in 2015 that were smear tested for bacterial resistance. The inclusion of resistance testing in the health facility would help in the surveillance and monitoring of patients' response to the anti-TB drugs.

## Conclusion

Over all, the treatment success rate was 81.2% and in keeping with national recommendation of 80% and above. As a trend, the treatment success rate though steady for the first three years, showed a decline in the last two years under study. There was no association seen between patient characteristics and TB treatment success in cases reviewed in this study.

## Recommendation

The observed increase in the proportion of cases that defaulted in the last two years of the study period with its likely effect on treatment success would require further research through a qualitative enquiry into the factors leading to default in these patients. The DOTS treatment center should strengthen its outreach services in the follow up of patients on treatment. Continued community health education to the populace on the need for TB patients to adhere to treatment and maintain linkage to the health facility committed to their care is also recommended.
